# Validation of Low-Cost IMUs for Telerehabilitation Exercises

**DOI:** 10.3390/s25103129

**Published:** 2025-05-15

**Authors:** Federico Caramia, Emanuele D’Angelantonio, Leandro Lucangeli, Valentina Camomilla

**Affiliations:** 1 Department of Movement, Human and Health Sciences, University of Rome “Foro Italico”, Piazza Lauro de Bosis 6, 00135 Rome, Italy; federico.caramia@uniroma4.it (F.C.); emanuele.dangelantonio@technoscience.it (E.D.); leandro.lucangeli@technoscience.it (L.L.); 2 Interuniversity Centre of Bioengineering of the Human Neuromusculoskeletal System, University of Rome “Foro Italico”, Piazza Lauro de Bosis 6, 00135 Rome, Italy; 3 Technoscience PST, Via Enrico Toti, 04100 Latina, Italy

**Keywords:** telemedicine, movement quality, movement intensity, physical exercises, inertial sensors, IoT

## Abstract

Telerehabilitation, a specialized domain within telemedicine, supports remote physical rehabilitation and progress monitoring. Wearable sensors can improve this service by providing reliable monitoring of movement parameters, offering objective information into patients’ rehabilitation sessions. This study presents the development and validation of a telerehabilitation system including a rehabilitation protocol, low-cost wearable inertial measurement units (IMUs) and a set of metrics descriptive of movement capacity to analyze rehabilitation exercises. Eleven medically stable elders (9 females, 2 males; age: 72.6 ± 5.0 years; height: 1.66 ± 0.09 m; mass: 67.8 ± 9.8 kg) performed 12 rehabilitation upper/lower limb and trunk exercises. Movement analysis was conducted using a prototypical IMU sensor and commercially available IMU as a reference. Each exercise was automatically segmented into single repetitions, from which selected metrics were computed. Bland–Altman analysis was performed to evaluate measurement agreement and consistency between the systems across all parameters. Results indicate acceptable measurement agreement for key rehabilitation metrics, including movement quantity, accelerations intensity, and movement smoothness. However, angular velocity and movement stability reveal technical limitations requiring refinement prior to clinical implementation. Balancing measurement reliability and affordability of telerehabilitation system remains a crucial factor to offer an effective service to individuals with diverse health conditions.

## 1. Introduction

A valuable resource for contemporary healthcare systems is telemedicine and its various subsections. Grown during the COVID-19 pandemic, telemedicine is a health service that has evolved through telecommunication and electronic information technologies, enabling remote communication between patients and health professionals. It includes a range of services, such as teleconsultation, which provides online consultations with patients; telemonitoring, which offers remote monitoring; and telerehabilitation, which facilitates remote physical and psychiatric rehabilitation [1,2]. Telerehabilitation services can achieve outcomes comparable to conventional rehabilitation methods, reducing muscle weakness, maintaining high levels of physical activity, and enhancing both functional capacity and the physical aspects of quality of life [2,3,4,5]. Another advantage of telerehabilitation is its ability to eliminate various barriers that can hinder patient compliance, such as the need to travel to a medical center or the effort associated with scheduling in-person appointments [5]. By allowing patients to engage their therapy from the comfort of their homes, these services not only promote more convenient and comfortable rehabilitation treatment but also significantly decrease hospitalization costs [6].

Previously, many telerehabilitation services relied on monitoring exercise programs through video calls, often lacking the ability to monitor movement and vital parameters [7]. To address this limitation, there is a growing trend towards the adoption of wearable sensors that can provide various biofeedback, thereby enhancing safety and effectiveness of telerehabilitation experiences. The use of wearable sensors has been becoming more and more popular among people with various health conditions, such as neurological, cardiac, and orthopedic diseases [8,9]. Feedback provided by wearables can provide real-time indicators of physiological stress thresholds, or of correct execution of movements through biomechanical parameters, preventing overexertion and allowing personalization of rehabilitation, thereby enhancing its effectiveness [10]. Physiological biofeedback is provided for example to monitor cardiac activity in patients with cardiovascular diseases, using electrocardiograms (ECGs) or sensors to measure oxygen saturation levels (SpO2) [10]. Biomechanical biofeedback, employed for patients with orthopedic and neurological rehabilitation needs, allows analyzing aspects such as posture control, muscle activation, and movement [10]. A comprehensive evaluation of the patient’s movements is crucial for effective telerehabilitation systems.

Within this category, inertial measurement unit (IMU) sensors are currently widespread [10,11,12,13,14] and gained positive feedback by clinicians regarding their application for monitoring rehabilitation movements [15]. In particular, clinicians appreciated the easy-to-use nature of this technology, and their ability to analyze and provide a report in a short period of time [15]. Due to their low cost, IMU sensors represent a promising solution for motion monitoring in telerehabilitation programs, making their adoption more accessible and feasible on a large scale.

Besides the basic repetition count, this technology can integrate video observation with information on the quality of the gestures performed, which cannot be fully captured through video alone during rehabilitation exercises. IMUs enable the measurement of key biomechanical parameters, such as range of motion and movement smoothness [11], providing therapists with detailed data on functional and movement limitations that may affect movement quality.

To increase the effectiveness and diffusion of telerehabilitation, it is crucial to develop comprehensive platforms that can support every stage of the process, from initial assessments to treatment and follow-up [16]. In this perspective, telerehabilitation infrastructures that integrate IMUs as a monitoring instrument for motor rehabilitation exercises are under development [17,18]. The main challenge remains the development of affordable and scalable technologies that can be tailored to the specific needs of individual users, without compromising utility or data quality. While various telerehabilitation systems exist [1,2], few effectively integrate evidence-based exercise protocols with strategic parameter selection and low-cost implementation, high usability, and relevant biomechanical feedback. This gap can limit the widespread adoption of sensor-based telerehabilitation, particularly among patients facing functional barriers to care.

The REHACT project (teleREHabilitation for respiratory and motor reACTivation exercises) addresses these limitations with a novel, integrative framework that combines low-cost IMUs, patient-centered rehabilitation protocols, and relevant metrics into a telerehabilitation infrastructure [19]. This framework can provide healthcare professionals with remote monitoring of patient movements during motor and respiratory rehabilitation exercises [19], balancing cost and technical efficiency while allowing patients’ engagement, a crucial factor for long-term adherence to rehabilitation programs.

Our scientific contribution focuses on structuring evidence-based protocols and carefully selecting biomechanical parameters that effectively represent movement capacities correlated with functional rehabilitation outcomes. The entire system is designed to be used with consumer-grade sensors, aligning with the principles of the “Internet of Things” (IoT), “I”nterconnecting among devices using “T”hings/tools in the service [20], making it accessible and practical in telerehabilitation context. The full implementation of the project requires the fulfillment of the following key objectives:

1. Development of an effective rehabilitation protocol, based on scientific literature and kinesiological expertise, which can be performed remotely through wearable sensors and provide biofeedback;
2. Selection of a set of parameters capable of describing different motor capacities;
3. Assessment of the validity of prototypical sensors;
4. Evaluation of the effectiveness of the IoT-based system in individuals with chronic conditions, identifying potential limitations;
5. Assessment of the system’s usability in a home environment.

Within this framework, the present study addresses the initial three objectives, focusing on the development of the rehabilitation protocol, the definition of relevant movement parameters, and the laboratory-based validation of the IMU prototypes under Technology Readiness Level 4 (TRL4) [21].

## 2. Materials and Methods

### 2.1. REHACT Motor Rehabilitation Protocol

**Figure 1:**
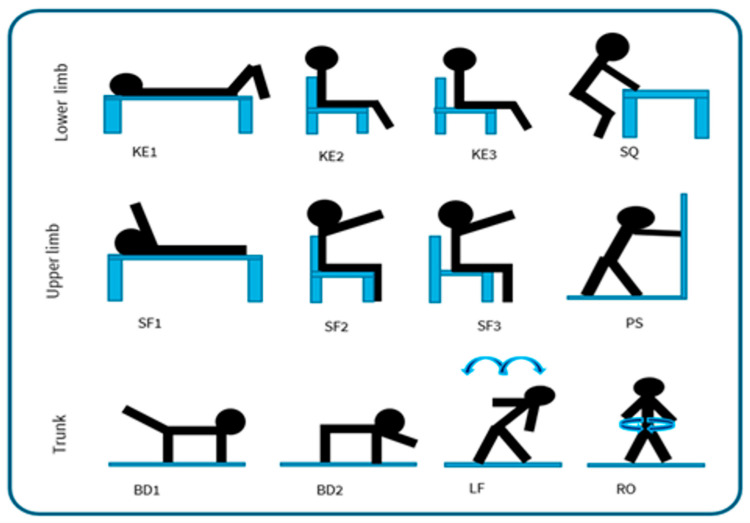
REHACT motor rehabilitation protocol. Lower limb exercises: KE1—knee extension lying down; KE2—knee extension sitting with a back support; KE3—knee extension without back support; SQ—half squat supported on a table. Upper limb exercises: SF1—shoulder flexion lying down; SF2—shoulder flexion sitting with back support; SF3—shoulder flexion without back support; PS—wall push-up. Trunk exercises: BD1—bird dog using only the legs; BD2—bird dog using only the arms; LF—lateral flexion of the column; RO—rotation of the column.

The “REHACT” motor rehabilitation protocol was developed based on a literature review and following clinical guidelines [22,23]. It includes twelve exercises selected according to a principle we named as “EASE”: Easy to perform for patients, Adaptable for different functional capacities, Safe and Effective to improve strength and mobility of the lower limbs, upper limbs, and trunk muscles. The main innovation consists of their integration with wearable inertial sensors that enable quantitative monitoring and remote feedback in real time. For example, the so-called “open chain” exercises [22,23] feature easy execution and low impact, while still providing a valuable rehabilitative effect and can be monitored with a small number of sensors, unlike more complex movements that would require a more elaborate setup. To adapt the protocol to individual functional capacity, the exercises can be performed in various positions: lying down, seated with back support, seated without back support, and standing with support. As shown in Figure 1, the resulting protocol includes:

- For the lower limbs: knee extension lying down (KE1), knee extension sitting with a back support (KE2), knee extension without back support (KE3), half squat supported on a table (SQ);
- For the upper limbs: shoulder flexion lying down (SF1), shoulder flexion sitting with a back support (SF2), shoulder flexion without back support (SF3), wall push-up (PS);
- For the trunk: bird dog exercise [24] using only the legs (BD1), bird dog using only the arms (BD2), lateral flexion of the column (LF), rotation of the column (RO).

**Figure 2:**
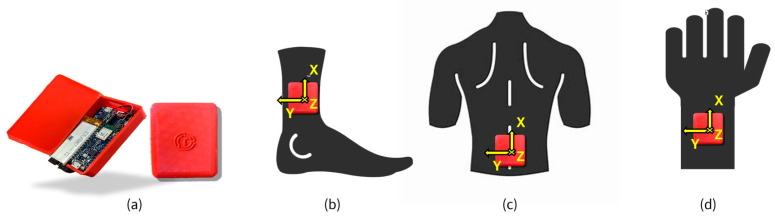
(**a**) Prototypical IMU, (**b**) positioning for the lower limb exercises, (**c**) positioning for trunk exercises, and (**d**) positioning for upper limb exercises.

To monitor the REHACT protocol, three sensors (Figure 2a) are required [19]: one placed on the lumbar region, and two positioned on the legs or arms depending on the specific exercise. The lumbar prototypical IMU is positioned at the lower back, secured with an elastic belt, except during exercises performed while lying down (Figure 2c). For exercises targeting lower limbs, the prototypes are attached to the participant’s tibias laterally above the malleolus and tightly secured with elastic straps within participants’ comfort tolerances (Figure 2b). For the upper limb exercises, the prototypes are attached just above the wrists (Figure 2d).

### 2.2. REHACT Sensors and Infrastructure

The REHACT infrastructure includes IMUs, which serve as data acquisition and transmission units, a mobile application, responsible for synchronizing and aligning the signals from the different IMUs ensuring accurate processing for motor gesture characterization, and an online database to store data collected via prototypes through the application.

Each prototypical sensor is equipped with a 9-axis IMU (LSM9DS1, STMicroelectronics, Plan-les-Ouates, Switzerland), which provides a full-scale range of ±4 g for the accelerometer, ±2000 deg/s for the gyroscope, and ±6 Gauss for the magnetometer, with a sampling frequency between 30–35 sample/s. The core of the system is the Arduino Nano BLE (Arduino S.r.l, Monza, Italy, dimensions: 45 × 18 mm, 64 MHz), which handles data acquisition and transmission via its integrated NINA B306 Bluetooth Low Energy module (u-blox AG, Thalwil, Switzerland). Power is supplied by three 3.7V LiPo batteries (620 math each), ensuring about 10 h of continuous use. Each battery weighs 12 g and measures 50 × 22.5 × 6.3 mm. All components, including a Step-Up module (XF0378X5) to boost voltage to 5V and a TP4056 charging board for USB recharging, are enclosed in a compact 3D-printed case (7 × 4.5 × 1.5 cm), ensuring stability, portability, and minimal user discomfort for a final mass of 35 g (Figure 2a).

**Figure 3:**
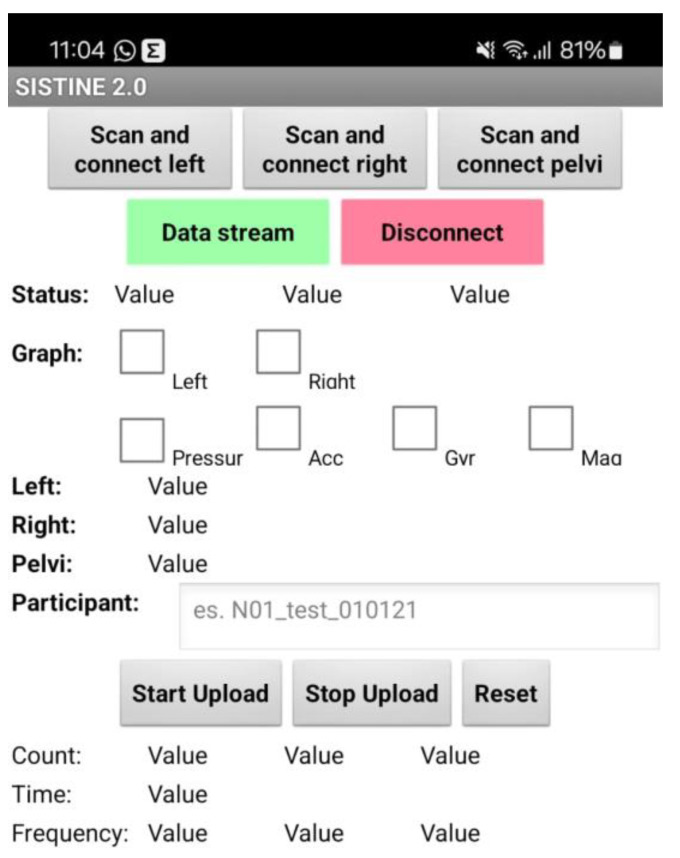
Screenshot of the smartphone app to manage the IMUs.

The Arduino Nano BLE facilitates the connection to a mobile application, developed using MIT App Inventor, version nb189 (© 2012–2024 Massachusetts Institute of Technology), that represents the transmission unit for the data collected during the exercises. The mobile application (Figure 3) securely stores data collected in a database (Firebase, Google, Mountain View, CA, USA).

Each user accesses the database through a unique identifier consisting of a username and password, which allows the data to be written and accessed. In addition, the database is accessible only to those with that identifier. Currently, for data analysis, the data are extracted from the database and analyzed using MATLAB R2021b (MathWorks Inc., Natik, MA, USA) and Google Colaboratory 3.11.12 (Google, Mountain View, CA, USA).

To ensure effective synchronization of the transmitted data, a software-based timing system was developed, designing a virtual clock to synchronize and coordinate the clocks of all IMUs before data transmission to the app and subsequently to the database. Through an iterative optimization process, different synchronization and coordination configurations were tested, achieving a data rate range of 30–35 samples per second for each sensor. This value was deemed adequate to monitor the REHACT rehabilitation protocol.

The same calibration procedure is applied at each session. For the gyroscope offset, the drift is automatically removed at each power-up by recording the signal while the device is held in a static position. For the accelerometer and magnetometer, calibration tests were conducted with the aim of identifying the specific biases and offsets. The values obtained from these calibrations were then used to correct the raw data before the analysis. This strategy was designed to facilitate the future use of the device in telerehabilitation settings, where the end user may not be able, or lacks the technical expertise, to perform calibration procedures themselves.

A key target in the development of the infrastructure is ensuring an optimal cost–benefit ratio. The developed sensor has an extremely low production cost (about thirty euros per unit). Even including the overall development costs, the technology is expected to be deployed at an affordable price for the individual user. This aspect would benefit the infrastructure, promoting its large-scale distribution to a large number of people performing telerehabilitation.

### 2.3. Movement Characterization

Movement rehabilitation exercises were characterized in terms of their quantity, intensity, and quality using the sensors.

**Figure 4:**
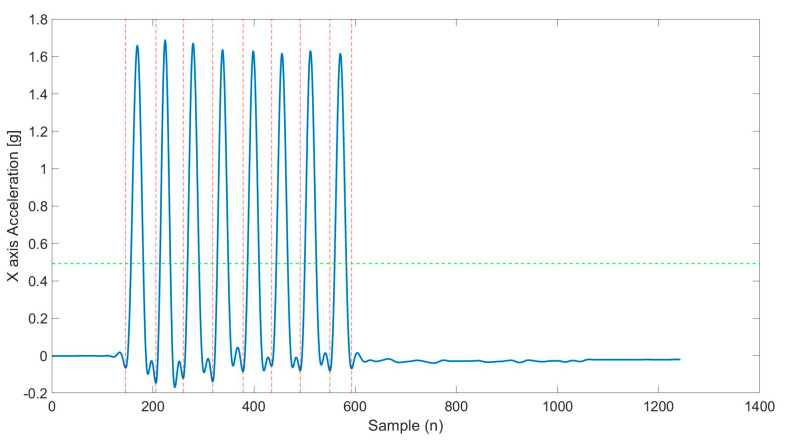
Example of repetition identification in the prototype’s data. The principal axis (in this case, the X-axis), is segmented in single repetitions (vertical red dashed lines). In green, the used threshold.

Data pre-processing was conducted to, first, low-pass filtering the signal, with an optimal cutoff frequency identified for each signal as in [25]. Data sets were then segmented into cycles, identifying start and end points of each repetition (Figure 4). A threshold of 30% of maximum peak acceleration values was selected following an iterative testing process with various cutoff values applied across all exercises in the protocol. This value proved to be the most effective in ensuring stable and reliable segmentation of movement cycles. Peaks related to the different exercises were detected along the axis where the movement of the analyzed exercise was most evident (e.g., antero-posterior direction, y-axis, for upper and lower limb exercises) using the “find peaks” function, whose settings were selected through an iterative testing process to identify the parameter combination that enabled the detection of the highest number of correct repetitions across the various exercises: “minimum peak width” = 0.2, “minimum peak prominence” = 0.1–0.2, and “minimum distance between peaks” = 30–50 samples (MATLAB, R2021, MathWorks Inc., Natik, MA, USA). For each detected peak, local minima below a predefined threshold were identified before and after the peak. The closest minimum before the peak was defined as the starting point, while the closest minimum after the peak marked the ending point. In the case of successive repetitions, the ending point of one movement coincided with the starting point of the next. These time points enabled the automatic segmentation of the repetition of different exercises.

The following features were selected as metrics to assess quantity, intensity, and quality of movements for individual repetitions or for each set of repetitions:

*Quantity* (QT), commonly utilized in traditional rehabilitation, includes fundamental aspects such as the number and duration of the repetitions, which were computed based on the identified cycle start and stop events:

- *REP*—number of repetitions made by the participant for each set;
- *TIME*—duration, for each repetition;

*Intensity* (I) of the movements, is associated to overall physical activity levels and described by parameters derived from the acceleration of body segments [11,12], which were complemented by peak values of measured signals:

- *Acceleration Peak* ($a_{X}^{peak}$, $a_{Y}^{peak}$, $a_{Z}^{peak}$)—the acceleration peaks on the different axes (X, Y, Z) were determined by extracting the maximum values within each identified repetition of the filtered data sets;
- *Angular velocity Peak* ($\omega_{X}^{peak}$, $\omega_{Y}^{peak}$, $\omega_{Z}^{peak}$)—angular velocity peaks about the different axes (X, Y, Z) were calculated as maximum value within each identified repetition of the filtered data sets;
- *Range of angular velocity* (RAV)—difference between maximum and minimum values of the Euclidean norm of the raw angular velocity [11,12] within each identified repetition.
- *Movement Intensity* (MI)—the mean value of the Euclidean norm of the linear triaxial filtered acceleration ($a_{x}\left(t\right),a_{y}\left(t\right),a_{z}\left(t\right)$) of the wearable sensor, as measured in g, was calculated over the exercise sets in healthy participant [11,12]. This metric was used in previous studies to quantify exercise intensity in clinical applications [12,26,27].
  (1) $$MI\left(t\right)=\frac{\sqrt{{a_{x}(t)}^{2}+{a_{y}(t)}^{2}+a_{z}{\left(t\right)}^{2}}}{g}$$
- *Movement intensity variation* (MIV)—difference in the MI values calculated for the two sets.

*Quality* of movements (QL), refers to the ability to perform actions in a controlled and optimal manner [28]. Joint range of motion, as suggested by [28], along with the smoothness and stability of gestures, as indicated by [12], were assess using the following parameters:

- *Log dimensionless jerk* (LDLJ)—measures gesture smoothness based on filtered acceleration data in each repetition [29,30], where smaller negative values correspond to smoother movements [12];
  (2) $$LDLJ=-\ln\left(\frac{t2-t1}{a_{peak}^{2}}\int_{t_{1}}^{t_{2}}\dddot{x}{\left(t\right)}^{2}+\dddot{y}{\left(t\right)}^{2}+\dddot{z}{\left(t\right)}^{2}dt\right)$$
  where $\dddot{x}{\left(t\right)}^{2}$, $\dddot{y}{\left(t\right)}^{2}$, $\dddot{z}{\left(t\right)}^{2}$ are the derivatives of the sensor’s triaxial acceleration with respect to time; $a_{peak}$ is equal to the magnitude of the peak total acceleration minus the mean total acceleration of the movement, and t1 and t2 represent the time at beginning and end of the repetition;
- *Dynamic Time Warping* (DTW)—assesses movement stability [11,27]. DTW was used to compare filtered acceleration signals from successive repetitions of the same set, with the DTW distance for each consecutive pair serving as an indicator of stability. This analysis was performed using the default “dtw” function provided in MATLAB. A lower DTW distance value can be interpreted as a better ability to maintain control of the joint movement while performing the exercise [12];
- *Peak range of Motion* (PKROM)—This index was used to assess the maximum range of motion [28]. First, the orientation of each sensor was computed for every repetition using the Madgwick orientation algorithm [31], whose beta coefficients were defined for each set of sensors as those minimizing orientation differences between devices, based on magnetometer calibration data (β prototype = 0.6, β reference = 0.1). Then, the joint angle (in degrees) was calculated along the specific axis of movement for each exercise (ROM), and finally, the peak values were computed within each identified repetition (PKROM).

### 2.4. Validation Study

The validity of the set of prototypical IMUs in monitoring quantitatively the quantity, intensity, and quality of the REHACT rehabilitation protocol exercises was assessed. Validation was performed, in terms of agreement and consistency, in comparison to a standard reference commercially available IMUs (Table 1), previously validated against a motion capture system [32] (OPAL, APDM, Portland, OR, USA). These transmit raw data wirelessly to a docking station, which synchronizes the signals and forwards them to a dedicated software for PC. The reference IMUs were placed over the prototypes, already secured on the participants’ body segments, fixing them with an additional elastic band similarly tightened to comfort tolerances. To minimize positioning-related errors, all sensors were placed by the same experimenter (F.C.). Table 1 summarizes the characteristics of the two different IMUs.

The study, approved by the University Research Committee (CAR code 158/2023), was conducted in accordance with the Helsinki Declaration as revised in 2024. Written informed consent was obtained from the participants of the study. Since this is a laboratory validation of the prototype, a convenience sample of 11 medically stable elderly participants (9 females and 2 males; age = 72.6 ± 5.0 years; height = 1.66 ± 0.09 m; mass = 68 ± 10 kg) were recruited. “Medically stable” means that the person’s health condition is managed, with normal vital parameters and no need for specific rehabilitation interventions. In fact, all participants performed the exercises without difficulty, or any musculoskeletal issue. Each participant had a Barthel Functional Index [33] score of 5, indicating their ability to function independently, and they had not experienced any falls in the past year [33]. Each participant performed the selected trunk, lower, and upper limb exercises for 2 sets of 8 repetitions each, with a recovery interval of 1 min between each set to avoid fatigue. The participants were asked to rate their perception of exertion (RPE) during the exercises using the Borg CR-10 Scale [34]. They were also asked to evaluate their comfort level while wearing the sensors using a Visual Analog Scale (VAS). This comprehensive approach allowed thorough assessment of both functional capabilities and subjective experiences of the participants during the validation process.

### 2.5. Statistical Analysis

Bland and Altman’s analysis [35] was applied to compare devices in terms of agreement by calculating for each parameter the BIAS (i.e., the mean difference between the values measured by the reference device and those of the prototype, useful for highlighting any systematic overestimates or underestimates) and the limits of agreement (LoA), defined as BIAS ± one standard deviation of the differences, which indicate the range within which most discrepancies between the two instruments are expected to fall, thus allowing an assessment of the degree of interchangeability or the presence of significant differences in their measurements. Outliers (>2 SD from the mean) were inspected for data set and excluded, to ensure reliable agreement assessment (see Appendix A). Subsequently, a possible heteroscedasticity of the data was verified via Kendall’s Tau test [36], by comparing the distribution of the averages with the absolute differences of the reference against prototype values [37]. If τ < 0.1, data are considered homoscedastic, i.e., having constant variance. Conversely, if τ ≥ 0.1, data are considered heteroscedastic, i.e., having variable variance. The 95% confidence intervals (CI) of BIAS and LoA, reported in Appendix A, were calculated as reported by [38]: t-value and standard error for the BIAS (seBIAS) used of CI calculations.

## 3. Results

The proposed exercises required minimal exertion from the participants, as indicated by the low RPE values (Table 2). The subjects experienced a high level of comfort when wearing the IMUs during the execution of rehabilitative exercises, as described by VAS values (Table 2).

A total of 176 repetitions were performed for each exercise. The automated algorithm, applied to both the prototype and the reference sensor, identified the same number of repetitions.

Table 3 presents the parameters values obtained from the IMUs.

**Figure 5:**
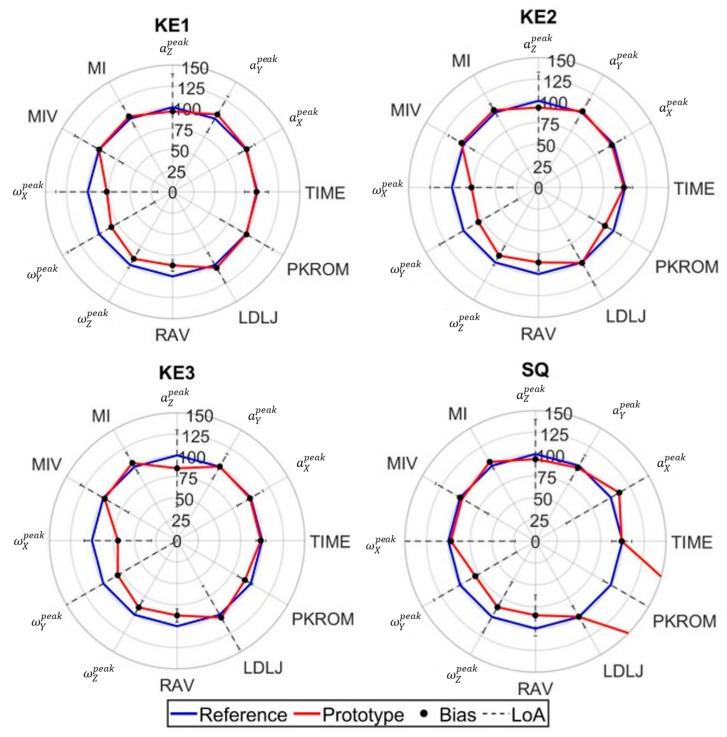
Percentage values of prototype parameters (red line) are reported compared to the reference values (100% line reported in blue). The black dot represents the mean of the differences between the two devices (Bias), in percentage of the reference value. The dashed line highlights the amplitude of the limits of agreement (LoA), in percentage the reference value. Values on each radar are relative to Intensity and Quality parameters: peak acceleration measurements ($a_{X}^{peak}$, $a_{Y}^{peak}$, $a_{Z}^{peak}$), peak angular velocity ($\omega_{X}^{peak}$, $\omega_{Y}^{peak}$, $\omega_{Z}^{peak}$), movement intensity and movement intensity variability (MI, MIV), range of angular velocity (RAV), log dimensionless jerk (LDLJ), and maximum value of range of motion (PKROM). Radars are provided for the following exercises: KE1—knee extension lying down; KE2—knee extension sitting with back support; KE3—knee extension without back support; SQ—half squat in support.

**Figure 6:**
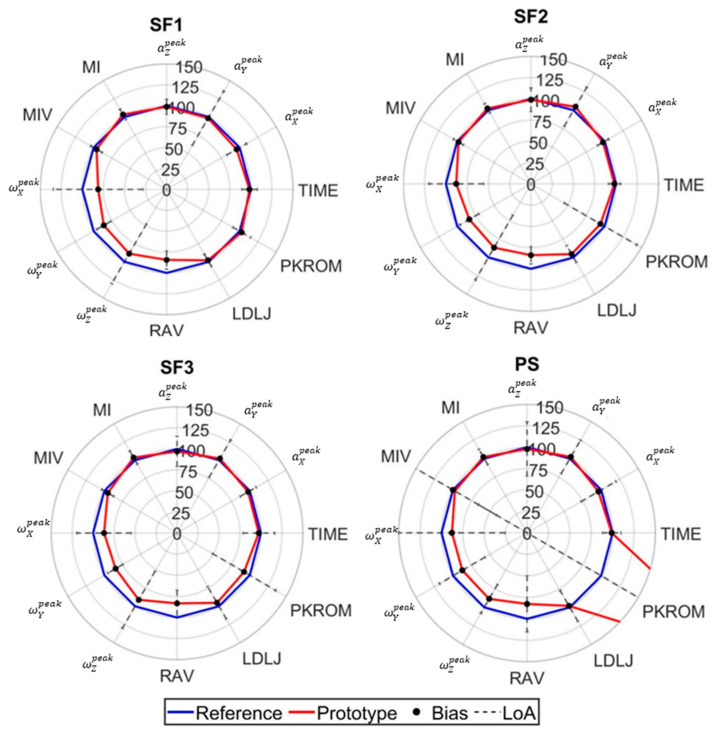
Percentage values of prototype parameters (red line) are reported compared to the reference values (100% line reported in blue). The black dot represents the mean of the differences between the two devices (Bias), in percentage of the reference value. The dashed line highlights the amplitude of the limits of agreement (LoA), in percentage the reference value. Values on each radar are relative to Intensity and Quality parameters: peak acceleration measurements ($a_{X}^{peak}$, $a_{Y}^{peak}$, $a_{Z}^{peak}$), peak angular velocity ($\omega_{X}^{peak}$, $\omega_{Y}^{peak}$, $\omega_{Z}^{peak}$), movement intensity and movement intensity variability (MI, MIV), range of angular velocity (RAV), log dimensionless jerk (LDLJ), and maximum value of range of motion (PKROM). Radars are provided for the following exercises: SF1—shoulder flexion lying down; SF2—shoulder flexion sitting with back support; SF3—shoulder flexion without back support; PS—wall push-up.

**Figure 7:**
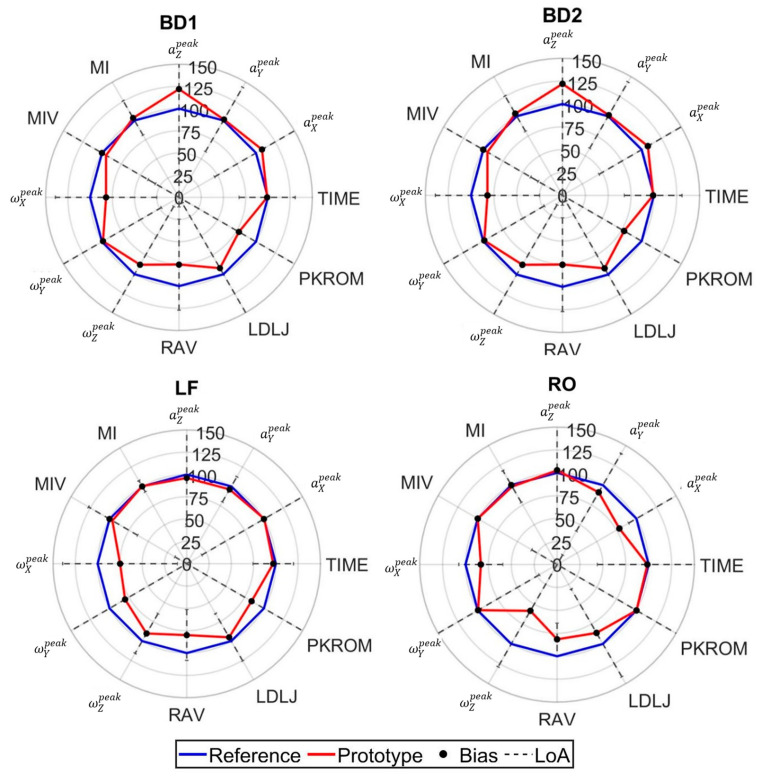
Percentage values of prototype parameters (red line) are reported compared to the reference values (100% line reported in blue). The black dot represents the mean of the differences between the two devices (Bias), in percentage of the reference value. The dashed line highlights the amplitude of the limits of agreement (LoA), in percentage the reference value. Values on each radar are relative to Intensity and Quality parameters: peak acceleration measurements ($a_{X}^{peak}$, $a_{Y}^{peak}$, $a_{Z}^{peak}$), peak angular velocity ($\omega_{X}^{peak}$, $\omega_{Y}^{peak}$, $\omega_{Z}^{peak}$), movement intensity and movement intensity variability (MI, MIV), range of angular velocity (RAV), log dimensionless jerk (LDLJ), and maximum value of range of motion (PKROM). Radars are provided for the following exercises: BD1—bird dog using only the legs; BD2—bird dog using only the arms; LF—lateral flexion of the column; RO—rotation of the column.

The difference between reference and prototypical IMUs sensors is demonstrated through radar plots (Figure 5, Figure 6 and Figure 7), based on the Bland and Altman analysis. In these radar plots, percentage values of prototype parameters are reported relative to the reference value, along with the bias and LoA between the two instruments. Quality parameter DTW was consistently heteroscedastic (Table A5) and was consequently excluded from Figure 5, Figure 6 and Figure 7. More details on the other BA parameters are reported in the Appendix A (Table A1, Table A2, Table A3, Table A4 and Table A5).

## 4. Discussion

The REHACT project’s development of an accessible and user-friendly telerehabilitation system based on the use of wearable sensors for movement characterization was structured around three objectives:

1. design a rehabilitation protocol, based on scientific evidence and kinesiological expertise, which could be performed remotely using wearable sensors;
2. select a set of parameters for motor capacity assessment;
3. validate prototype sensors against reference systems.

The REHACT rehabilitation protocol, developed through literature analysis and clinical expertise, demonstrated strong usability metrics when tested on medical stable elderly, supporting its potential implementation in real-world settings. Participants reported very low perceived exertion scores while performing the exercises (RPE 0.8–2.1), despite some individual variability possible due to different fitness level, and perceived the prototype sensors as comfortable (VAS 7.85–9.09), despite the simple attachment system using elastic bands.

An algorithm for automatic repetition identification, tailored through threshold selection to each exercise in the protocol, was successfully implemented across both sensor types, enabling consistent biomechanical metrics extraction and direct comparison between prototype and reference systems. This approach facilitates movement characterization and establishes a parameter set applicable in remote monitoring contexts.

The implementation of low-cost IMUs for motor exercise monitoring represents a useful step towards accessible telerehabilitation solutions, within the REHACT project and in general. The systematic evaluation of the prototype sensors against established commercial systems, across multiple parameters, highlights both the limitations and potential of these cost-effective monitoring solutions in the perspective of their clinical viability.

Consistency of the prototype and its agreement with reference sensors are commented on in the perspective of highlighting which parameter types can be used in an applicative setting:

- Quantity parameters exhibited robust validity for conventional rehabilitation parameters (repetition count and execution time) (Figure 5, Figure 6 and Figure 7), demonstrating homoscedastic behavior across all evaluated exercises (τ < 0.1) (Table A1). The system validity is further substantiated by the use of the same algorithm for repetition identification in both reference and prototype sensors. This consistency is a basic prerequisite for reliable monitoring physical therapy exercises.
- Movement Intensity parameters showed varying degrees of consistency and of agreement (Figure 5, Figure 6 and Figure 7). Open chain exercises (KE1, KE2, KE3, SF1, SF2, SF3) had a better performance in terms of both BIAS and LoA with respect to closed chain ones (SQ, PS, BD1, BD2, LF, RO). While peak acceleration measurements ($a_{X}^{peak}$, $a_{Y}^{peak}$, $a_{Z}^{peak}$) and derived parameters (MI, MIV) demonstrated good consistency and agreement, angular velocity measurements ($\omega_{X}^{peak}$, $\omega_{Y}^{peak}$, $\omega_{Z}^{peak}$)showed substantial variability and disagreement. For example, in the lower limb exercises (Table A2), biases range from −0.48 to −16.9, while the limits of agreement (LoA) vary between 4.7 and 26.0 across the different exercises. Similar patterns can be observed for both upper limb (Table A3) and trunk exercises (Table A4). Although these differences appear to be reduced in the RAV parameter, they highlight the need for hardware improvement.
- Quality parameters had different behaviors: LDLJ showed acceptable consistency; conversely, DTW had a heteroscedastic behavior, which led to its exclusion and calls for a refinement in the stability assessment; PKROM had high LoA in most of the exercises, presenting a limited applicability for rehabilitation environment. For example, in the SQ exercise, the prototype estimated a PKROM of 29 degrees, while in the PS exercise, it measured 65 degrees, which is significantly higher compared to the reference sensor values (Table 3). Acceptable levels of consistency and variability can be observed in the KE2, KE3, and SF1 exercises, together with reasonably narrow limits of agreement and the favorable cost-benefit ratio of the prototype, suggesting the use of PKROM as suitable for monitoring in practical applications. The error in orientation estimation certainly suffers from the abovementioned differences amongst angular velocities, not excluding other sources of error and calling for implementing ad hoc sensor fusion algorithms.

This laboratory assessment confirms the prototype’s potential for evaluating certain movement parameters during a rehabilitation protocol. Besides repetition count and execution time, Intensity parameters related to acceleration proved to be the most consistent, and plausibly acceptable for use in rehabilitation environments (LoA in general below 10%). High values of MI indicate phases of rapid accelerations and decelerations, previously resulting in values close to 1 for lower limb exercises performed by a young population [12]. Our sample presented lower values, particularly for lower limb and trunk exercises (Table 3). MI values can be used as a monitoring tool, since values close to zero, i.e., a very low intensity, are desirable for rehabilitation exercises that require great control of the joint and attention on the part of the subject. Among Quality parameters, the obtained LDLJ values, despite a higher variability, constitute reference values for movement quality during rehabilitation exercises. Specifically, medically stable elderly participants showed values between −1.4 and −1.9 (Table 3). In comparison, LDLJ values between −3 and −10 are reported in the literature for upper limb exercises performed by patients post-stroke [30]. This is coherent with the interpretation of the metric: high LDLJ denotes reduced smoothness, while those closer to zero reflect smoother movements.

The prototypes’ capability to measure certain movement parameters offers useful support to rehabilitation protocols. With an estimated production cost of approximately 30 euros per sensor (Table 1), the system could represent an affordable solution for remote monitoring, particularly beneficial for individual users.

### Limits and Future Development

Several limitations may have affected the consistency between the two systems. Firstly, hardware and firmware differences (Table 1) potentially influenced the computation of certain parameters, such as PKROM, angular velocities or DTW. Additionally, software-related aspects, for example the prototypical sensors calibration, likely contributed to discrepancies in the results. Dedicated metrological tests are still essential to improve the results obtained and to evaluate hardware/firmware and software improvements. Specific tests on angular velocity measurements may more clearly show the gyroscope drift trend over time, with possible improved correction at firmware level or development and implementation of prototype-specific calibration algorithms. In addition, the introduction of firmware/software solutions that increase the sampling rate could lead to better results, constituting important future development.

Secondly, overlapping and fixing reference and prototype sensors in the same position could also have influenced the consistency between the two systems, despite being necessary to compare location-dependent parameters. Besides the contingent overlap, consistent and reliable measurements of the prototype require robust sensor attachment methods and clear user guides, particularly for when patients will apply the sensors independently.

Thirdly, the inclusion of exclusively medically stable participants limits external validity restricting generalizability of the findings to clinical populations. The sample size potentially limited statistical power of the validation process. Moreover, controlled laboratory settings (TRL4), may inadequately reflect real-world conditions or clinical scenarios. Future research should include a broader range of medically stable elderly subjects as well as conduct real-word validation (TRL5) with people with chronic disease to confirm generalizability of the findings and evaluate parameters’ sensitivity to detect clinically meaningful changes in motor performance, thereby enabling more precise assessment of improvement or potential deterioration in motor skills execution.

Lastly, implementation of an algorithm for automatic exercise recognition, potentially integrating data from all three sensors currently included, would substantially enhance the safety features of the system by detecting both non-adherence to the rehabilitative indication and incorrect movements performed by the patient. This advancement could further improve autonomy of use and adaptability of the system, particularly within telerehabilitation frameworks.

## 5. Conclusions

This research presents an affordable telerehabilitation framework through the REHACT system, advancing the field through a novel combination of evidence-based exercise protocols, strategic parameter selection, and low-cost IMU technology, bringing closer the potentially conflicting requirements of cost-effectiveness, ease of use, and clinical relevance. Effective biomechanical monitoring does not necessitate high-cost equipment when paired with cautious selection of established analysis methods.

Despite some technical limitations, the prototype IMUs demonstrated consistent results across several rehabilitation parameters, including gesture smoothness, movement intensity, and movement quantity. Incorporating these parameters into remote rehabilitation platforms could significantly enhance patient monitoring, enable personalized and adaptive rehabilitation plans, and support clinical decision-making.

This study lays the technological groundwork for a telerehabilitation system designed to be both affordable and usable to real-world healthcare needs. In particular, its low cost compared to commercial alternatives makes it a promising candidate for large-scale adoption in home-based rehabilitation.

Future phases of the REHACT project will expand to evaluating system usability in real-life contexts and assessing long-term clinical outcomes, with the overarching goal of increasing treatment adherence and enhancing the quality of life for patients through affordable, effective, and evidence-based remote healthcare solutions.

## Figures and Tables

**Table 1:** Characteristics of prototypical inertial sensor and reference sensor. * Average estimate.

Characteristics	Prototypical IMU	Reference IMU
Unit cost	≈30 euros (production cost)	Up to 2000 euros * (commercial cost)
Dimension	7 × 4.5 × 1.5 cm	5 × 4 × 1.4 cm
Mass	35 g	26 g
Battery	10 h	Up to 12 h
Connection	Bluetooth	Wireless
Sample frequency	32 Hz	128 Hz
Accelerometer range	±4 g	±6 g
Gyroscope range	±2000 deg/s	±2000 deg/s
Magnetometer range	±6 Gauss	±6 Gauss

**Table 2:** VAS and RPE results reported for all exercises grouped in categories. Lower limb exercises: KE1—knee extension lying down; KE2—knee extension sitting with a back support; KE3—knee extension without back support; SQ—half squat supported on a table. Upper limb exercises: SF1—shoulder flexion lying down; SF2—shoulder flexion sitting with back support; SF3—shoulder flexion without back support. Trunk exercises: PS—wall push-up; BD1—bird dog using only the legs; BD2—bird dog using only the arms; LF—lateral flexion of the column; RO—rotation of the column. RPE values are expressed in arbitrary units, VAS ones on a scale 0–10.

Category	Exercises	RPE [a.u.]	VAS [0–10]
Lower limb	KE1	1.4 ± 1.5	9.1 ± 1.2
	KE2	1.3 ± 1.1	9.1 ± 1.2
	KE3	0.8 ± 1.0	9.1 ± 1.2
	SQ	0.9 ± 1.0	9.1 ± 1.2
Upper limb	SF1	1.2 ± 1.6	8.7 ± 1.5
	SF2	1.5 ± 1.6	8.7 ± 1.5
	SF3	1.8 ± 1.8	8.7 ± 1.5
	PS	1.4 ± 1.1	8.7 ± 1.5
Trunk	BD1	1.4 ± 1.5	9.1 ± 1.2
	BD2	2.1 ± 1.8	7.9 ± 3.1
	LF	1.1 ± 1.2	8.8 ± 1.4
	RO	1.8 ± 1.8	8.7 ± 1.6

**Table 3:** Parameter values (mean and standard deviation) as assessed using the reference IMUs and the prototype IMU are reported for all exercises grouped in categories. Lower limb exercises: KE1—knee extension lying down; KE2—knee extension sitting with a back support; KE3—knee extension without back support; SQ—half squat supported on a table. Upper limb exercises: SF1—shoulder flexion lying down; SF2—shoulder flexion sitting with back support; SF3—shoulder flexion without back support. Trunk exercises: PS—wall push-up; BD1—bird dog using only the legs; BD2—bird dog using only the arms; LF—lateral flexion of the column; RO—rotation of the column.

**Reference IMUs**																
**Category**	**Exercises**		**Quantity**		**Intensity**									**Quality**		
			**REP**	**TIME** **[s]**	$a_{X}^{peak}$ **[g]**	$a_{Y}^{peak}$ **[g]**	$a_{Z}^{peak}$ **[g]**	$\omega_{X}^{peak}$ **[deg/s]**	$\omega_{Y}^{peak}$ **[deg/s]**	$\omega_{Z}^{peak}$ **[deg/s]**	**MI** **[g]**	**MIV** **[g]**	**RAV ** **[rad/s]**	**LDLJ** **[a.u.]**	**PKROM** **[deg]**	**DTW** **[m/s^2^]**
Lower limb	KE1	Mean	8	2.3	1.6	0.5	0.3	26.0	43.4	197.7	0.3	0.2	0.5	−1.6	117.4	4.6
		*Std*	*0*	*0.5*	*0.2*	*0.1*	*0.2*	*13.1*	*27.6*	*27.9*	*0*	*0*	*0.1*	*0.2*	*14.4*	*2.2*
	KE2	Mean	8	1.8	0.9	0.8	0.2	32.2	48.9	205.2	0.3	0.1	0.6	−1.5	75.2	7.5
		*Std*	*0*	*0.5*	*0.2*	*0.1*	*0.1*	*18.4*	*37.8*	*41.5*	*0*	*0*	*0.1*	*0.6*	*13.5*	*3.4*
	KE3	Mean	8	1.6	0.8	0.7	0.2	32.5	42.1	197.3	0.2	0.1	0.4	−1.5	73.2	6.6
		*Std*	*0*	*0.4*	*0.2*	*0.1*	*0*	*10.2*	*30.1*	*44.8*	*0*	*0*	*0.1*	*0.7*	*10.3*	*3.6*
	SQ	Mean	8	2.3	0.1	0.4	0.1	6.6	11.9	45.3	0.2	0	0.1	−1.9	9.2	3.2
		*Std*	*0*	*0.6*	*0.1*	*0.1*	*0.1*	*3.9*	*7.7*	*12.3*	*0*	*0*	*0*	*0.2*	*3.5*	*2.8*
Upper limb	SF1	Mean	8	3.2	0.7	0.8	2.0	52.6	181.0	69.4	0.8	0.6	0.5	−1.8	153.9	8.2
		*Std*	*0*	*1.1*	*0.2*	*0.4*	*0.4*	*26.8*	*39.9*	*41.7*	*0.1*	*0.1*	*0.1*	*0.2*	*28.1*	*5.5*
	SF2	Mean	8	2.5	1.4	0.7	1.1	72.3	188.0	54.9	0.7	0.4	0.5	−1.9	69.1	6.8
		*Std*	*0*	*0.6*	*0.3*	*0.2*	*0.4*	*27.3*	*36.6*	*28.3*	*0.1*	*0.1*	*0.1*	*0.3*	*11.1*	*4.8*
	SF3	Mean	8	2.2	1.5	0.7	0.9	82.7	195.9	68.4	0.6	0.3	0.5	−1.9	66.9	7.2
		*Std*	*0*	*0.6*	*0.2*	*0.3*	*0.4*	*36.8*	*45.2*	*40.0*	*0.1*	*0*	*0.1*	*0.4*	*13.3*	*6.0*
	PS	Mean	8	2.6	0.4	0.2	0.2	12.4	35.7	44.4	0.2	0	0.1	−1.4	22.3	4.8
		*Std*	*0*	*0.5*	*0.2*	*0.1*	*0.1*	*9.3*	*18.5*	*10.6*	*0.1*	*0*	*0*	*0.6*	*12.3*	*4.6*
Trunk	BD1	Mean	8	2.0	0.1	0.2	0	21.8	5.7	8.7	0.1	0	0.1	−1.3	29.4	4.9
		*Std*	*0*	*0.5*	*0.1*	*0.1*	*0*	*10.8*	*3.5*	*3.7*	*0.1*	*0*	*0*	*1.0*	*17.2*	*5.4*
	BD2	Mean	8	1.9	0.1	0.1	0.1	6.4	10.9	6.5	0.1	0	0	−0.9	65.7	2.1
		*Std*	*0*	*0.5*	*0.1*	*0.1*	*0.1*	*4.5*	*6.6*	*4.1*	*0*	*0*	*0*	*0.9*	*17.5*	*2.2*
	LF	Mean	8	2.8	0	0.2	0.1	10.9	5.7	15.4	0.1	0	0	−1.6	63.5	10.0
		*Std*	*0*	*0.8*	*0*	*0.1*	*0*	*5.9*	*3.8*	*8.5*	*0*	*0*	*0*	*0.5*	*26.4*	*6.7*
	RO	Mean	8	2.6	0	0.1	0.1	80.5	7.0	20.7	0.1	0	0.2	−2.0	52.9	3.5
		*Std*	*0*	*0.7*	*0*	*0*	*0*	*19.3*	*3.3*	*11.3*	*0*	*0*	*0*	*0.7*	*22.1*	*2.3*
**Prototype IMUs**																
**Category**	**Exercises**		**Quantity**		**Intensity**									**Quality**		
			**REP**	**TIME** **[s]**	$a_{X}^{peak}$ **[g]**	$a_{Y}^{peak}$ **[g]**	$a_{Z}^{peak}$ **[g]**	$\omega_{X}^{peak}$ **[deg/s]**	$\omega_{Y}^{peak}$ **[deg/s]**	$\omega_{Z}^{peak}$ **[deg/s]**	**MI** **[g]**	**MIV** **[g]**	**RAV** **[rad/s]**	**LDLJ** **[a.u.]**	**PKROM** **[deg]**	**DTW** **[m/s^2^]**
Lower limb	KE1	Mean	8	2.3	1.5	0.5	0.2	21.1	35.2	179.7	0.3	0.2	0.4	−1.6	117.2	2.1
		*Std*	*0*	*0.5*	*0.1*	*0*	*0.1*	*13.1*	*22.2*	*27*	*0*	*0*	*0*	*0.2*	*19.9*	*1.4*
	KE2	Mean	8	1.7	0.8	0.8	0.2	25.4	38.2	186.5	0.2	0.1	0.4	−1.5	66.5	2.7
		*Std*	*0*	*0.4*	*0.1*	*0.1*	*0.1*	*16.3*	*29.4*	*42.4*	*0*	*0*	*0.1*	*0.6*	*10.7*	*2.6*
	KE3	Mean	8	1.6	0.8	0.7	0.2	22.5	32.1	177.3	0.2	0.1	0.4	−1.5	63.2	2.6
		*Std*	*0*	*0.4*	*0.2*	*0.1*	*0*	*10.2*	*20.1*	*34.8*	*0*	*0*	*0.1*	*0.7*	*9.3*	*1.6*
	SQ	Mean	8	2.3	0.1	0.1	0.5	6.5	35.5	3.5	0.2	0	0.1	−1.9	21.0	1.3
		*Std*	*0*	*0.5*	*0*	*0*	*0.1*	*3.2*	*165*	*1.8*	*0*	*0*	*0*	*0.2*	*8.4*	*1.1*
Upper limb	SF1	Mean	8	3.1	0.6	0.7	1.9	42.2	155.6	60.2	0.8	0.6	0.3	−1.8	160.6	3.3
		*Std*	*0*	*1.5*	*0.2*	*0.3*	*0.4*	*20.2*	*35.6*	*33*	*0.1*	*0.1*	*0*	*0.2*	*24.1*	*1.7*
	SF2	Mean	8	2.4	1.3	0.7	1.1	63.5	157.2	47.3	0.7	0.4	0.4	−1.9	66.8	2.5
		*Std*	*0*	*0.5*	*0.2*	*0.2*	*0.3*	*24.3*	*29.5*	*23.6*	*0*	*0.1*	*0*	*0.3*	*15.2*	*1.1*
	SF3	Mean	8	2.1	1.4	0.7	0.8	71.8	165.4	61.7	0.6	0.3	0.4	−1.9	63.2	3.3
		*Std*	*0*	*0.5*	*0.2*	*0.2*	*0.3*	*32.1*	*38.9*	*34.3*	*0.1*	*0*	*0.1*	*0.4*	*15.3*	*3.9*
	PS	Mean	8	2.5	0.1	0.1	0.3	4.6	18.4	1.4	0.1	0	0.1	−1.4	34.9	0.6
		*Std*	*0*	*0.4*	*0*	*0*	*0.1*	*2.2*	*7.8*	*0.8*	*0*	*0*	*0*	*0.6*	*17.3*	*0.5*
Trunk	BD1	Mean	8	2.0	0.1	0.2	0.1	17.8	5.8	7.9	0.1	0	0	−1.3	23.5	1.4
		*Std*	*0*	*0.5*	*0*	*0*	*0*	*8.4*	*3.5*	*4.6*	*0*	*0*	*0*	*1.0*	*13.3*	*1.5*
	BD2	Mean	8	1.8	0.1	0.1	0.1	6	8.8	5.6	0.1	0	0	−0.9	69.1	0.5
		*Std*	*0*	*0.4*	*0*	*0*	*0*	*3.7*	*5.4*	*4*	*0*	*0*	*0*	*0.9*	*15.5*	*0.4*
	LF	Mean	8	2.6	0	0.2	0.1	8.2	4.5	13.8	0.1	0	0	−1.6	53.6	1.3
		*Std*	*0*	*0.8*	*0*	*0*	*0*	*4.4*	*2.5*	*7.5*	*0*	*0*	*0*	*0.5*	*26.2*	*2.0*
	RO	Mean	8	2.5	0	0.1	0.1	66.9	7.4	12	0.1	0	0.1	−2.0	56.3	0.7
		*Std*	*0*	*0.7*	*0*	*0*	*0*	*17.3*	*4*	*5.5*	*0*	*0*	*0*	*0.7*	*27.8*	*0.5*

## Data Availability

The raw data supporting the conclusions of this article will be made available by the authors on request.

## Abbreviations

The following abbreviations are used in this manuscript:

ECG	Electrocardiogram
SpO_2_	Oxygen saturation levels
IMU	Inertial measurement unit
IoT	Internet of Things
TRL	Technology Readiness Level
KE1	Knee extension lying down
KE2	Knee extension sitting with back support
KE3	Knee extension sitting without back support
SQ	Half squat in support
SF1	Shoulder flexion lying down
SF2	Shoulder flexion sitting with back support
SF3	Shoulder flexion sitting without back support
PS	Wall push up
BD1	Bird dog using legs
BD2	Bird dog using arms
LF	Trunk lateral flexion
RO	Trunk Rotation
QT	“Quantity” movement parameter
REP	Number of repetitions
TIME	Time to perform repetition
I	“Intensity” movement parameter
$a_{X}^{peak}$	Peak of acceleration x-axis
$a_{Y}^{peak}$	Peak of acceleration y-axis
$a_{Z}^{peak}$	Peak of acceleration z-axis
$\omega_{X}^{peak}$	Peak of angular velocity x-axis
$\omega_{Y}^{peak}$	Peak of angular velocity y-axis
$\omega_{Z}^{peak}$	Peak of angular velocity z-axis
MI	Movement intensity
MIV	Movement intensity variability
RAV	Range of angular velocity
QL	“Quality” movement parameter
LDLJ	Log dimensionless jerk
DTW	Dynamic time warping
ROM	Range of motion
PKROM	Maximum value of range of motion
RPE	Rate of perception exertion
VAS	Visual analog scale
BIAS	Difference between the mean reference and the
LoA	Limit of agreement
BA	Bland and Altman analysis
CI	Confidence intervals
seBIAS	Standard error for the BIAS

## Appendix A

### Bland and Altman Parameters

For Bland and Altman’s analysis conducted on the two sensors, Table A1, Table A2 and Table A3 show the parameter values of Bias, LoA, %CI Bias, %Ci LoA, Kendall’s τ, Samples, t-value, and seBIAS.

**Table A1:** Bland and Altman analysis for quantity parameters in terms of: Bias, LoA (all expressed in the measurement units indicated for each parameter), %CI Bias, %CI LoA and τ, number of outliers removed from the 176 repetitions identified, t-value, and seBIAS. Data are reported for all exercises. Lower limb exercises: KE1—knee extension lying down; KE2—knee extension sitting with a back support; KE3—knee extension without back support; SQ—half squat supported on a table. Upper limb exercises: SF1—shoulder flexion lying down; SF2—shoulder flexion sitting with back support; SF3—shoulder flexion without back support; PS—wall push-up. Trunk exercises: BD1—bird dog using only the legs; BD2—bird dog using only the arms; LF—lateral flexion of the column; RO—rotation of the column.

Exercises	Parameter	Bias	LoA	CI% Bias	CI% LoA	Kendal’s τ	Outliers	t-Value	se Bias
KE1	REP	0	0	0%	0%	\	0	0	0
	TIME [s]	0.02	0.32	1%	3%	0.01	6	1.96	0
KE2	REP	0	0	0%	0%	\	0	0	0
	TIME [s]	0.02	0.17	0%	1%	0.01	0	1.96	0
KE3	REP	0	0	0%	0%	\	0	0	0
	TIME [s]	0.02	0.10	0%	1%	0.01	0	1.96	0
SQ	REP	0	0	0%	0%	\	0	0	0
	TIME [s]	0.02	0.25	10%	2%	0.06	4	1.96	0
SF1	REP	0	0	0%	0%	\	0	0	0
	TIME [s]	−0.05	0.26	1%	2%	0.01	8	1.96	0
SF2	REP	0	0	0%	0%	\	0	0	0
	TIME [s]	−0.04	0.17	0%	1%	0.05	5	1.96	0
SF3	REP	0	0	0%	0%	\	0	0	0
	TIME [s]	−0.05	0.41	2%	3%	0.01	2	1.96	0.01
PS	REP	0	0	0%	0%	\	0	0	0
	TIME [s]	−0.02	0.27	1%	2%	0.01	8	1.96	0
BD1	REP	0	0	0%	0%	\	0	0	0
	TIME [s]	−0.01	0.63	4%	7%	0.09	0	1.96	0.02
BD2	REP	0	0	0%	0%	\	0	0	0
	TIME [s]	−0.01	0.35	2%	4%	0.07	5	1.97	0.01
LF	REP	0	0	0%	0%	\	0	0	0
	TIME [s]	−0.03	0.61	11%	20%	0.07	10	1.97	0.05
RO	REP	0	0	0%	0%	\	0	0	0
	TIME [s]	−0.03	1.40	11%	2%	0.07	10	1.97	0.05

**Table A2:** Statistical analysis for the intensity parameters in terms of: Bias, LoA (all expressed in the measurement units indicated for each parameter), %CI Bias, %CI LoA and τ, number of outliers removed from the 176 repetitions identified, t-value, and seBias. Data are reported for the lower limb exercises: KE1—knee extension lying down; KE2—knee extension sitting with a back support; KE3—knee extension without back support; SQ—half squat supported on a table.

Exercises	Parameter	Bias	LoA	CI% Bias	CI% LoA	Kendal’s τ	Outliers	t-Value	se Bias
KE1	$a_{X}^{peak}$[g]	0.01	0.16	0%	1%	0.01	3	1.96	0
	$a_{Y}^{peak}$[g]	0.02	0.12	0%	1%	0.05	4	1.96	0
	$a_{Z}^{peak}$[g]	0.01	0.11	0%	1%	0.01	7	1.96	0
	$\omega_{X}^{peak}$[deg/s]	−5.4	14.7	0.8	13%	0.06	7	1.96	0.4
	$\omega_{Y}^{peak}$[deg/s]	−5.9	21.0	1.19	20%	0.03	19	1.96	0.6
	$\omega_{Z}^{peak}$[deg/s]	−16.9	24.7	1.32	22%	0.04	0	1.96	0.6
	MI [g]	0	0.04	0%	1%	0.05	0	2.01	0
	MIV [g]	0	0.06	1%	1%	0.06	0	2.01	0
	RAV [rad/s]	0.06	0.03	0%	0%	0.05	1	1.96	0
KE2	$a_{X}^{peak}$[g]	0.02	0.15	0%	1%	0.08	0	1.96	0
	$a_{Y}^{peak}$[g]	0.01	0.12	0%	1%	0.01	2	1.96	0
	$a_{Z}^{peak}$[g]	0.01	0.09	0%	0%	0.02	9	1.96	0
	$\omega_{X}^{peak}$[deg/s]	−6.9	14.6	79%	13%	0.08	5	1.96	0.4
	$\omega_{Y}^{peak}$[deg/s]	−7.7	20.1	11%	19%	0.07	15	1.96	0.5
	$\omega_{Z}^{peak}$[deg/s]	−18.6	26.3	14%	23%	0.01	0	1.96	0.7
	MI [g]	0	0.02	0%	0%	0.03	0	2.01	0
	MIV [g]	0	0.01	0%	0%	0.03	0	2.01	0
	RAV [rad/s]	0.07	0.05	0%	0%	0.05	0	1.96	0
KE3	$a_{X}^{peak}$[g]	0.04	0.11	0%	0%	0.02	0	1.96	0
	$a_{Y}^{peak}$[g]	0.01	0.12	0%	0%	0.02	0	1.96	0
	$a_{Z}^{peak}$[g]	0.01	0.10	0%	0%	0.02	0	1.96	0
	$\omega_{X}^{peak}$[deg/s]	−7.0	13.6	59%	11%	0.08	5	1.96	0.5
	$\omega_{Y}^{peak}$[deg/s]	−7.1	18.1	10%	16%	0.05	4	1.96	0.3
	$\omega_{Z}^{peak}$[deg/s]	−13.6	24.5	14%	23%	0.01	0	1.96	0.2
	MI [g]	0	0.02	0%	0%	0.03	0	2.01	0
	MIV [g]	0	0.01	0%	0%	0.03	0	2.01	0
	RAV [rad/s]	0.06	0.04	0%	0%	0.01	0	1.96	0
SQ	$a_{X}^{peak}$[g]	0.01	0.06	0%	0%	0.03	19	1.96	0
	$a_{Y}^{peak}$[g]	0.01	0.07	0%	0%	0.02	15	1.96	0
	$a_{Z}^{peak}$[g]	0	0.05	0%	0%	0.08	20	1.96	0
	$\omega_{X}^{peak}$[deg/s]	0.18	4.7	26%	46%	0.02	15	1.96	0.13
	$\omega_{Y}^{peak}$[deg/s]	−2.2	6.0	34%	59%	0.02	22	1.96	0.17
	$\omega_{Z}^{peak}$[deg/s]	−5.8	15.2	9%	14%	0.02	15	1.96	0.43
	MI [g]	0	0.01	0%	0%	0.01	2	2.02	0
	MIV [g]	0	0	0%	0%	0.01	3	2.02	0
	RAV [rad/s]	0.02	0.03	0%	0%	0.01	14	1.96	0

**Table A3:** Statistical analysis for the intensity parameters in terms of: Bias, LoA (all expressed in the measurement units indicated for each parameter), %CI Bias, %CI LoA and τ, number of outliers removed from the 176 repetitions identified, t-value, and seBias. Data are reported for the upper limb exercises: SF1—shoulder flexion lying down; SF2—shoulder flexion sitting with back support; SF3—shoulder flexion sitting without back support; PS—wall push up * Heteroscedastic parameters.

Exercises	Parameter	Bias	LoA	CI% Bias	CI% LoA	Kendal’s τ	Outliers	t-Value	se Bias
SF1	$a_{X}^{peak}$[g]	−0.02	0.09	0%	0%	0.01	7	1.96	0
	$a_{Y}^{peak}$[g]	−0.01	0.28	1%	2%	0.01	2	1.96	0
	$a_{Z}^{peak}$[g]	−0.02	0.18	1%	1%	0.04	3	1.96	0
	$\omega_{X}^{peak}$[deg/s]	−9.0	25.5	14%	24%	0.02	10	1.96	0.7
	$\omega_{Y}^{peak}$[deg/s]	−25.2	17.5	9%	16%	0.02	1	1.96	0.47
	$\omega_{Z}^{peak}$[deg/s]	−6.2	23.0	13%	22%	0.02	13	1.96	0.67
	MI [g]	0.02	0.06	1%	1%	0.01	2	2.02	0
	MIV [g]	−0.02	0.05	0%	1%	0.08	0	2.01	0
	RAV [rad/s]	−0.07	0.05	0%	0%	0.01	0	1.96	0
SF2	$a_{X}^{peak}$[g]	−0.03	0.10	0%	1%	0.01	6	1.96	0
	$a_{Y}^{peak}$[g]	0.03	0.17	0%	1%	0.01	3	1.96	0
	$a_{Z}^{peak}$[g]	−0.01	0.14	0%	1%	0.06	6	1.96	0
	$\omega_{X}^{peak}$[deg/s]	−8.8	23.2	12%	21%	0.02	0	1.96	0.63
	$\omega_{Y}^{peak}$[deg/s]	−30.7	24.6	13%	22%	0.04	0	1.96	0.66
	$\omega_{Z}^{peak}$[deg/s]	−6.6	22.0	12%	20%	0.02	0	1.96	0.61
	MI [g]	0.01	0.04	0%	1%	0.05	1	2.01	0
	MIV [g]	0	0.02	0%	0%	0.02	2	2.02	0
	RAV [rad/s]	−0.07	0.04	0%	0%	0.05	0	1.96	0
SF3	$a_{X}^{peak}$[g]	−0.03	0.16	0%	1%	0.01	6	1.96	0
	$a_{Y}^{peak}$[g]	0.01	0.17	0%	1%	0.08	3	1.96	0
	$a_{Z}^{peak}$[g]	−0.02	0.15	0%	1%	0.01	6	1.96	0
	$\omega_{X}^{peak}$[deg/s]	−10.2	25.2	13%	24%	0.02	0	1.96	0.63
	$\omega_{Y}^{peak}$[deg/s]	−30.4	20.4	10%	18%	0.04	0	1.96	0.66
	$\omega_{Z}^{peak}$[deg/s]	−5.26	23.1	12%	22%	0.02	8	1.96	0.61
	MI [g]	0.02	0.05	0%	1%	0.04	1	2.01	0
	MIV [g]	−0.01	0.03	0%	0%	0.01	2	2.02	0
	RAV [rad/s]	−0.09	0.08	0%	0%	0.6 *	0	1.96	0
PS	$a_{X}^{peak}$[g]	−0.01	0.08	0%	0%	0.04	23	1.96	0
	$a_{Y}^{peak}$[g]	0	0.05	0%	0%	0.01	25	1.96	0
	$a_{Z}^{peak}$[g]	0	0.04	0%	0%	0.03	30	1.96	0
	$\omega_{X}^{peak}$[deg/s]	−1.3	6.6	39%	68%	0.01	36	1.96	0.2
	$\omega_{Y}^{peak}$[deg/s]	−4.4	10.7	62%	107%	0.04	48	1.96	0.3
	$\omega_{Z}^{peak}$[deg/s]	−5.1	8.7	50%	87%	0.01	24	1.96	0.25
	MI [g]	0	0.02	0%	0%	0.08	3	2.02	0
	MIV [g]	0	0.01	0%	0%	0.01	3	2.02	0
	RAV [rad/s]	−0.02	0.04	0%	0%	0.01	25	1.96	0

**Table A4:** Statistical analysis for the intensity parameters in terms of: Bias, LoA (all expressed in the measurement units indicated for each parameter), %CI Bias, %CI LoA and τ, number of outliers removed from the 176 repetitions identified, t-value, and seBias. Data are reported for the upper limb exercises: BD1—bird dog exercise using only the legs; BD2—bird dog exercise using only the arm; LF—lateral flexion; RO—trunk rotation. * Heteroscedastic parameters.

Exercises	Parameter	Bias	LoA	CI% Bias	CI% LoA	Kendal’s τ	Outliers	t-Value	se Bias
BD1	$a_{X}^{peak}$[g]	0	0.04	0%	0%	0.01	8	1.97	0
	$a_{Y}^{peak}$[g]	0	0.1	0%	1%	0.08	0	1.96	0
	$a_{Z}^{peak}$[g]	0	0.04	0%	0%	0.01	6	1.97	0
	$\omega_{X}^{peak}$[deg/s]	−3.9	12.4	78%	135%	0.03	0	1.96	0.39
	$\omega_{Y}^{peak}$[deg/s]	0	3.4	21%	37%	0.02	7	1.97	0.11
	$\omega_{Z}^{peak}$[deg/s]	−1	6.3	40%	69%	0.01	2	1.96	0.2
	MI [g]	0	0.03	0%	1%	0.01	0	2.1	0
	MIV [g]	0	0	0%	0%	0.02	1	2.1	0
	RAV [rad/s]	−0.01	0.02	0%	0%	0.02	4	1.96	0
BD2	$a_{X}^{peak}$[g]	0	0.03	0%	0%	0.01	6	240	0
	$a_{Y}^{peak}$[g]	0	0.03	0%	0%	0.01	1	179	0
	$a_{Z}^{peak}$[g]	0	0.05	0%	0%	0.02	2	208	0.01
	$\omega_{X}^{peak}$[deg/s]	−0.3	3.7	24%	43%	0.02	2	224	0.39
	$\omega_{Y}^{peak}$[deg/s]	−1.9	3.4	22%	38%	0.04	2	231	0.37
	$\omega_{Z}^{peak}$[deg/s]	−0.9	2.8	19%	33%	0.03	3	209	0.06
	MI [g]	0	0.01	0%	0%	0.03	0	18	0.15
	MIV [g]	0	0	0%	0%	0.03	0	16	0.07
	RAV [rad/s]	0	0	0%	0%	0.04	0	221	0.3
LF	$a_{X}^{peak}$[g]	0	0.01	0%	0%	0.03	12	1.97	0
	$a_{Y}^{peak}$[g]	0	0.03	0%	0%	0.09	12	1.97	0
	$a_{Z}^{peak}$[g]	0	0.03	0%	0%	0.02	13	1.97	0
	$\omega_{X}^{peak}$[deg/s]	−13.5	19.4	15%	27%	0.06	11	1.97	0.8
	$\omega_{Y}^{peak}$[deg/s]	−0.0	5.2	43%	75%	0.01	16	1.97	0.22
	$\omega_{Z}^{peak}$[deg/s]	−8.6	14.6	11%	20%	0.5 *	12	1.97	0.6
	MI [g]	0	0	0%	0%	0.03	1	2.1	0
	MIV [g]	0	0	0%	0%	0.01	0	2.1	0
	RAV [rad/s]	−0.09	0.08	0%	0%	0.6 *	0	1.96	0
RO	$a_{X}^{peak}$[g]	−0.03	1.40	0%	0%	0.03	12	1.97	0
	$a_{Y}^{peak}$[g]	0	0.01	0%	0%	0.09	12	1.97	0
	$a_{Z}^{peak}$[g]	0	0.03	0%	0%	0.02	13	1.97	0
	$\omega_{X}^{peak}$[deg/s]	0	0.0	115%	274%	0.06	11	1.97	0.8
	$\omega_{Y}^{peak}$[deg/s]	−13.5	19.4	43%	75%	0.01	16	1.97	0.22
	$\omega_{Z}^{peak}$[deg/s]	−0.0	5.2	119%	206%	0.5 *	12	1.97	0.6
	MI [g]	−8.61	14.56	0%	0%	0.03	1	2.1	0
	MIV [g]	0	0	0%	0%	0.01	0	2.1	0
	RAV [rad/s]	−0.03	0.03	0%	0%	0.02	12	1.97	0

**Table A5:** Statistical analysis for the quality parameters in terms of: Bias, LoA (all expressed in the measurement units indicated for each parameter), %CI Bias, %CI LoA and τ, number of outliers removed from the 176 repetitions identified, t-value, and seBias. Data are reported for the lower limb exercises: KE1—knee extension lying down; KE2—knee extension sitting with a back support; KE3—knee extension without back support; SQ—half squat supported on a table. Data are also reported for the upper limb exercises: SF1—shoulder flexion lying down; SF2—shoulder flexion sitting with back support; SF3—shoulder flexion without back support; PS—wall push-up. Data are also reported for the trunk exercises: BD1—bird dog using only the legs; BD2—bird dog using only the arms; LF—lateral flexion of the column; RO—rotation of the column. * Heteroscedastic parameters.

Exercises	Parameter	Bias	LoA	CI% Bias	CI% LoA	Kendal’s τ	Outliers	t-Value	se Bias
KE1	LDLJ [a.u.]	0.06	0.32	1%	3%	0.01	0	1.96	0
	PKROM [deg]	0.7	41.9	23%	40%	0.05	40	1.96	0.09
	DTW [m/s^2^]	−1.9	3.1	2%	3%	0.06	4	1.96	1.15
KE2	LDLJ [a.u.]	0.00	0.48	2%	4%	0.03	7	1.96	0.01
	PKROM [deg]	−8.9	15.9	9%	15%	0.01	4	1.96	0.43
	DTW [m/s^2^]	−2.5	5.0	30%	53%	0.06	41	1.96	0.15
KE3	LDLJ [a.u.]	0.00	0.42	1%	2%	0.01	2	1.96	0.01
	PKROM [deg]	−8.9	11.9	6%	13%	0.01	4	1.96	0.43
	DTW [m/s^2^]	−2.0	4.8	30%	50%	0.03	40	1.96	0.15
SQ	LDLJ [a.u.]	0	0.27	1%	2%	0.01	30	1.96	0
	PKROM [deg]	20.9	21.8	12%	21%	0.7 *	14	1.96	0.62
	DTW [m/s^2^]	−1.7	2.6	20%	30%	0.6 *	44	1.96	0.08
SF1	LDLJ [a.u.]	0.03	0.15	0%	1%	0.08	1	1.96	0
	PKROM [deg]	4.7	20.4	13%	23%	0.0	54	1.96	0.67
	DTW [m/s^2^]	−4.3	6.5	40%	70%	0.7 *	34	1.96	0.19
SF2	LDLJ [a.u.]	0.08	0.21	1%	1%	0.01	4	1.96	0
	PKROM [deg]	−4.0	34.9	19%	33%	0.0	4	1.96	0.96
	DTW [m/s^2^]	−3.5	4.8	30%	50%	0.7 *	33	1.96	0.14
SF3	LDLJ [a.u.]	0.08	0.41	2%	3%	0.08	1	1.96	0.01
	PKROM [deg]	−5.4	30.1	160%	290%	0.0	7	1.96	0.83
	DTW [m/s^2^]	−3.1	5.6	30%	60%	0.6 *	47	1.96	0.17
PS	LDLJ [a.u.]	0.03	0.31	1%	3%	0.07	272	1.96	0
	PKROM [deg]	43.6	79.6	470%	82%	0.6 *	39	1.96	2.4
	DTW [m/s^2^]	−2.6	3.8	20%	40%	0.8 *	55	1.97	0.15
BD1	LDLJ [a.u.]	0.12	1.29	8%	14%	0.01	61	1.97	0.04
	PKROM [deg]	−5.8	33.4	216%	374%	0.03	54	1.96	1.09
	DTW [m/s^2^]	−2.3	4.2	29%	50%	0.7 *	73	1.97	0.14
BD2	LDLJ [a.u.]	0.11	0.92	6%	10%	0.03	58	1.97	0.03
	PKROM [deg]	2.3	25.0	166%	288%	0.03	61	1.97	0.84
	DTW [m/s^2^]	−1.0	1.5	11%	19%	0.7 *	78	1.97	0.05
LF	LDLJ [a.u.]	0.08	0.5	4%	7%	0.06	13	1.97	0.02
	PKROM [deg]	−9.8	60.7	506%	877%	0.02	18	1.97	2.56
	DTW [m/s^2^]	−2.3	4.2	41%	72%	0.8 *	36	1.98	0.21
RO	LDLJ [a.u.]	0.29	1.15	9%	16%	0.07	16	1.97	0.04
	PKROM [deg]	0.1	70.0	58%	1014%	0.02	17	1.97	2.96
	DTW [m/s^2^]	−2.3	3.13	28%	49%	0.7 *	27	1.97	0.14
